# Activation of the hypothalamic-pituitary adrenal axis in response to a verbal fluency task and associations with task performance

**DOI:** 10.1371/journal.pone.0227721

**Published:** 2020-04-16

**Authors:** Linda Becker, Ursula Schade, Nicolas Rohleder

**Affiliations:** Department of Psychology, Friedrich-Alexander University Erlangen-Nürnberg, Erlangen, Germany; Grenoble Faculty of Medicine and Hospital, FRANCE

## Abstract

Speech fluency can be impaired in stressful situations. In this study, it was investigated whether a verbal fluency task by itself, i.e. without the presence of any further stressors, induces responses of the hypothalamic-pituitary adrenal (HPA) axis and of the sympathetic nervous system (SNS). The sample consisted of *n* = 85 participants (68.2% female; 33.3 ± 15.2 years) who performed two consecutive verbal fluency tasks for two minutes each. The categories were either ‘stress’ or ‘disease’ and ‘animals’ or ‘foods’ which were presented in a randomized order. Three saliva samples were collected, prior to the task (t_0_), immediately after (t_1_), and ten minutes after it (t_2_). Salivary α-amylase and cortisol were assessed. Furthermore, blood pressure, heart rate, and ratings of actual stress perception, level of effort, and tiredness were measured. The verbal fluency task induced a HPA axis response with a maximum cortisol level at t_2_ which was independent of task performance. Furthermore, perceived stress and effort, as well as tiredness increased after the task. Moreover, tiredness immediately after the task was negatively correlated with task performance. No α-amylase, blood pressure, or heart rate, and therefore SNS, responses were found. Implications for the integrated specificity model are discussed. We conclude that a verbal fluency task acts like an acute stressor that induces a cortisol and a perceived stress response without the need for further (e.g., social-evaluative) stress components. Therefore, it is a less time-consuming alternative to other stress tasks that can be used in field studies with little effort.

## Introduction

Acute stress triggers a variety of physiological responses. The most prominent is the activation of the hypothalamus-pituitary adrenal (HPA) axis which leads to secretion of the stress hormone cortisol from the adrenal cortex [1,2]. Furthermore, the sympathetic nervous system (SNS) becomes activated in response to acute stressors which leads to the release of epinephrine and norepinephrine from the adrenal medulla, as well as to a variety of secondary reactions such as an increase in blood pressure and heart rate, and a decrease in heart rate variability [3–5].

Both stress systems can interact with the brain via direct and indirect pathways and can, therefore, alter brain chemistry which can consecutively alter cognitive functioning [6–8]. For example, retrieval from declarative long-term memory decreases after an acute stressor and this is associated with the HPA axis response [9–11]. Furthermore, working (or short-term) memory is affected in response to acute stressors which is associated with both the HPA axis and the SNS response [12–15]. Overviews about the associations between acute stress and cognitive functioning can be found in [16–19].

A cognitive function that has been less investigated so far, is verbal fluency (VF). At least two variants can be distinguished: first, semantic fluency (i.e., naming as many words as possible that belong to a specific category), and second phonemic fluency (i.e., naming as many words as possible that are beginning with a given letter). Verbal fluency in general requires a variety of cognitive processes such as long-term memory and working memory as well as executive functions [20,21]. Impairments in verbal fluency are often associated with frontal (phonemic and semantic fluency) or temporal (semantic fluency) brain lesions [22]. Therefore, verbal fluency tasks (VFT) are standard procedures in neuropsychological assessments.

It has been found previously that speech productivity can be impaired in acute stress situations. Buchanan, Laures-Gore, and Duff (2014; [23]) found that participants with high cortisol responses to the Trier Social Stress-Test (TSST; [24]) paused more during the stress task (and accordingly had a poorer VF performance) than participants with low cortisol responses. However, it cannot be deduced from these findings whether the poor VF performance was a result of the stress induction or whether the poor performance by itself led to an (additional) HPA axis response. Therefore, the aim of the present study was to investigate whether performing a VF task by itself, without any further stress induction, induces a stress response. If one was found, we were further interested in whether the stress response was associated with cognitive performance during the task. We hypothesized that a poor VF performance would be associated with a strong stress response. We decided to investigate semantic (and not phonemic) fluency and used a VFT in which as many words as possible that belong to a specific category should be named.

However, cognitive stressors are a special case of stressors because they do not threaten life but do indeed mobilize resources to face the task demands. The specific role of transmitted messenger molecules (e.g., stress hormones) is still unclear, though. Furthermore, it is discussed whether pure cognitive stressors trigger the same general stress response (i.e., an unspecific increase in SNS and HPA axis activity) or whether the stress response is specific and depends on the stressor characteristics, i.e. whether the stressor is, for example, cognitive, social-evaluative, or threatening [25,26]. Therefore, we differentiated between the activation of different stress systems: first, we investigated the perceived stress by the participants by means of rating scales, because it has been shown that stress perception can differ from physiological stress responses [27]. Second, reactivity of the HPA axis which was assessed using salivary cortisol measures. Third, the activation of the SNS which was measured by means of blood pressure, heart rate, and salivary alpha-amylase (sAA) assessments. The latter (sAA) has been shown to be a suitable non-invasive marker of SNS activation after acute stressors [28].

Furthermore, we aimed to investigate–if a response was found–whether this is associated with anthropometric and health factors (e.g., age, sex, body-mass index (BMI), depression, and use of oral contraceptives) that are typically related with HPA axis and SNS responses when other (usually social-evaluative) stress tasks are used (e.g., [29–35]). Since this was not the main research question of our study, we did not formulate specific hypotheses and investigated this exploratively instead. However, in order to achieve this goal, a heterogeneous sample was needed which was recruited from the general population.

## Materials and methods

### Participants

From initially *n* = 101 (67/101 = 66.3% female; 34.9 ± 15.2 years; BMI = 23.7 ± 4.0 kg/m^2^; 81/101 = 80.2% non-smokers) participants, ten (10/101 = 9.9%) were excluded because they did not provide enough saliva for analysis, five (5/101 = 5%) because German was not their mother tongue, and one (1/101 = 1%) because his performance in the VFT was below three standard deviations from the sample’s mean. The final sample consisted of *n* = 85 healthy participants (58/85 = 68.2% female; 33.3 ± 15.2 years, min.: 18, max.: 69; BMI = 23.7 ± 4.3 kg/m^2^, min.: 18.2 kg/m^2^, max.: 41.5 kg/m^2^; 71/85 = 83.5% non-smokers). Exclusion criteria were: usage of beta-blockers or glucocorticoid medication.

Most (62/85 = 72.9%) of the participants reported that they are regularly engaged in exercise on 3 ± 1.5 days per week on average. According to the cut-off value by Stein and Luppa (2012; [36]), ten participants (10/85 = 11.8%) were classified as depressive. Thirty-eight (38/85 = 44.7%) participants were classified as low and 47/85 (55.3%) as high-chronically stressed. An overview of all relevant descriptive sample characteristics is provided in Tables 1 and 2.

**Table 1:** Sample characteristics (mean, standard deviation, minimum, maximum) of the metric variables.

Variable	N	Mean	Standard deviation	Minimum	Maximum
Age	85	33.3	15.2	18	69
BMI	85	23.7	4.3	18.2	41,5
Sport days per week	61	3.0	1.6	0	7
PSS scale	85	15.4	6.8	2	36
CES-D scale	80	14.2	9.0	2	55

BMI: body-mass index; PSS: Perceived Stress Scale; CES-D: Center for Epidemiological Studies-Depression scale.

**Table 2:** Sample characteristics of the categorial variables.

Variable	Label	Number	Percentage
Sex	Female	58	68.2
	Male	27	31.8
BMI classification	Underweight	2	2.4
	Normal weight	61	71.8
	Pre-obese	15	17.6
	Obese	7	8,2
Marital Status	Single	39	45.9
	Married	23	27.1
	Partnership	21	24.7
	Divorced	1	1.2
	Widowed	1	1.2
Education	Certificate of secondary education ('Hauptschulabschluss')	4	4.7
	Secondary school level ('Mittlere Reife')	11	12.9
	Vocational diploma ('Fachabitur')	5	5.9
	General qualification for university entrance ('Abitur')	35	41.2
	Bachelor‘s degree	13	15.3
	Diploma or master‘s degree	11	12.9
	PhD	5	5.9
	Habilitation	1	1.2
Profession	Unemployed	2	2.4
	Trainee	5	5.9
	Student	31	36.5
	Employed	36	42.4
	Self-employed	1	1.2
	Official	6	7.1
	Pension	2	2.4
	Other	2	2.4
Smoking	Yes, daily	7	8.2
	Yes, sometimes	7	8.2
	No	71	83.5
Alcohol consumption on study day	Yes, but less than 2 beverages and not within the last 2 hours	11	12.9
	No	74	87.1
General alcohol consumption	No alcohol consumption	10	11.8
	1 day per week	16	18.8
	2 days per week	11	12.9
	3 days per week	6	7.1
	4 days per week	1	1.2
	5 and more days per week	3	3.5
	Less than 1 day per week	38	44.7
Regular sports participation	No	23	27.1
	Yes	62	72.9
Sports classification	Endurance sports	36	42.4
	Strength training	3	3.5
	Relaxation training	3	3.5
	Endurance and strength training	16	18.8
	Endurance and relaxation training	4	4.7
	Endurance, strength, and relaxation training	1	1.2
Use of oral contraceptives (women only)	No	47	81
	Yes	11	19
Perceived Stress Scale classification	Low-chronically stressed	38	44.7
	High-chronically stressed	47	55.3

An a-priori power analysis was performed, using G*Power (version 3.1.9.2). It indicated an optimal sample size of *n*_optimal_ = 82. Power analysis was performed for an analysis of variance (ANOVA) for repeated measurements (rmANOVA) with an α-level of α = .05, a power of 1 - β = .95, a medium effect size of *f* = 0.2 for the main effect of the variable ‘time’, three groups (e.g., for the ratings), and three measurement time points (t_0_, t_1_, and t_2_). The dependent variables were either sAA, cortisol, mean arterial pressure (MAP), heart rate, or perceived stress levels. Therefore, we assume that the achieved sample size of *n* = 85 is sufficient for the reported analyses.

All participants gave their written and informed consent. Data was anonymized directly after collection to protect participant’s privacy. The study was approved by the data protection commission of the Friedrich-Alexander University Erlangen-Nürnberg (FAU). The study was conducted according to the principles expressed in the Declaration of Helsinki and was approved by the local ethics committee of the FAU (# 397_19 B).

### Materials

#### Verbal fluency task

A semantic fluency task was used for assessing verbal fluency. The participants were given a category for which they should name as many terms as possible. Each participant was assigned two categories, one neutral and one emotional, and they were given two minutes time for each. The neutral category was either “animals” or “foods” as it is used in classical VF tasks (e.g., [37]). The emotional categories were either “stress” or “disease” which were developed by the authors themselves and which are not part of standard neuropsychological test batteries. By including this category, we intended to induce negative emotions which potentially intensifies the stress reaction compared to a pure neutral VFT. However, it was not our aim to compare the physiological reactions between emotional and neutral VF tasks. The order of the categories was counterbalanced between the participants. Participants were voice recorded during the VFT, using a digital dictaphone (Olympus VN-541PC). For VFT evaluation, the number of correct terms (VFT_corr,i_), number of repetitions (VFT_rep,i_), and number of other errors (VFT_oth,i_, e.g. wrong category) were determined and a performance score VFT_perf,i_ = VFT_corr,i_−(VFT_rep,i_ + VFT_oth,i_) was calculated for each category i = {1, 2}. From these, a mean performance score VFT_perf_ = (VFT_perf,1_ + VFT_perf,2_)/2 was calculated. Note: raw values instead of standard values were used for the VFT evaluation, because of the self-developed categories.

#### Rating scales

During each saliva sampling (at t_0_, t_1_, and t_2_), perceived stress, level of effort, and tiredness were rated on 10-point Likert scales by the participants. The participants were asked “*How stressed do you feel at this moment*?”, “*How strong is your level of effort at this moment*?”, and “*How tired do you feel at this moment*?”. The anchors were “*not stressed at all*” and “*extremely stressed*”, “*no effort*” and “*extreme effort*”, as well as “*not tired*” and “*extremely tired*”.

#### Saliva sampling and analysis

Saliva samples were collected by means of salivettes (Sarstedt, Nümbrecht, Germany). Participants were instructed to keep the salivette in their mouth for at least one minute and to move it back and forth, but not to chew on it. Saliva samples were stored at -30°C after collection for later analyses. Immediately before analysis, samples were centrifuged at 2000 g and 20°C for ten minutes. The same saliva samples were used for assessment of sAA and cortisol. From each saliva sample, 40 μl (2 * 20 μl) were taken for cortisol and 20 μl (2 * 10 μl) for sAA assessment. Salivary α-amylase was measured with an in-house enzyme kinetic assay using reagents from DiaSys Diagnostic Systems GmbH (Holzheim, Germany), as previously described [38,39]. In brief, saliva was diluted at 1:625 with ultrapure water and diluted saliva was incubated with substrate reagent (α-amylase CC FS; DiaSys Diagnostic Systems) at 37° C for three minutes before a first absorbance reading was taken at 405 nm with a Tecan Infinite 200 PRO reader (Tecan, Crailsheim, Germany). A second reading was taken after five minutes incubation at 37°C and increase in absorbance was transformed to sAA concentration (U/ml), using a standard curve prepared using “Calibrator f.a.s.” solution (Roche Diagnostics). Salivary cortisol concentrations were determined in duplicate using chemiluminescence immunoassay (CLIA, IBL, Hamburg, Germany). Intra- and inter-assay coefficients of variation were below 10% for both sAA and cortisol.

#### Blood pressure and heart rate

Systolic and diastolic blood pressure as well as heart rate were assessed by means of an upper arm blood-pressure monitor (boso medicus X). Two participants (2/85 = 2.4%) were excluded from blood pressure and heart rate analysis because of technical problems. Twenty-five participants (25/85 = 29.4%) were classified as hypertonic because their blood pressure during the t_0_ measurement was ≥140/90 mmHg. From these, only two (2/85 = 8%) reported a hypertension diagnosis. The hypertonic participants were initially excluded from analysis of the blood pressure and heart rate time course, but further analyses were performed that confirmed that the same effects were found for hypertonic and non-hypertonic participants. Since blood pressure might be related to the other physiological variables as well and, therefore, with the sAA or cortisol response, we investigated whether the same sAA and cortisol time courses were found for hypertonic and non-hypertonic participants. However, no differences were found as well (see below). For analysis of the blood pressure time course in response to the VFT, MAP was calculated as MAP = diastole + 0.412 * (systole–diastole) [40].

#### Demographic variables, health status, and lifestyle factors

Self-developed questionnaires were used to assess demographic variables (age, sex, height, weight, marital status, educational level, occupation, monthly income), health status (physiological and psychological diseases, medication) and lifestyle factors (smoking, alcohol consumption, participation in regular sports). The medication variable was used to control whether the exclusion criteria (no usage of beta-blockers or glucocorticoids) were fulfilled which was the case for all included participants. Furthermore, a new variable ‘use of oral contraceptives’ was derived for the female participants. Body-mass index was calculated from height and weight according to BMI = (weight [kg])/(height [m])^2^ and was classified according to the norms provided by the World Health Organization as underweight (< 18.5 kg/m^2^), normal weight (18.5–24.9 kg/m^2^), pre-obese (25–29.9 kg/m^2^), and obese (> 29.9 kg/m^2^).

#### Depression

Depression was assessed by means of the German version of the long form of the depression scale from the Center for Epidemiological Studies (CES-D; [41,42]). A cut-off value of 22 was used for classification into depressed and non-depressed participants [36]. The range of values of this scale is between 0 and 60. Cronbach’s α was α = .89 in our study.

#### Perceived life stress/ chronic stress

The perceived level of life stress within the last four weeks was assessed by means of a validated German translation of the 10-item version of the Perceived Stress Scale (PSS; [43,44]). Cut-off values of 13 for women (all ages), 12 for men younger than 40 years, and 13 for men equal to or above 40 years were used to differentiate between low-chronically stressed and high-chronically stressed participants [44]. The range of values of the PSS is between 0 and 40. Cronbach’s α was α = .88 in our study. Note: although the PSS assesses perceived stress within the last month and not explicitly chronic stress, we refer to it as a measure of chronic stress in the following to avoid confusion with our acute perceived stress measurement.

#### Affect

As last part of the questionnaire battery and immediately before the last saliva sample, actual affect was assessed by means of a German version of the Positive and Negative Affect Schedule (PANAS; [45–47]). The PANAS contains 20 adjectives (10 positive and 10 negative) based on which the current affective state should be rated. Cronbach’s α was α = .94 for positive affect and α = .87 for negative affect in our study.

### Procedure

About half of the participants (48/85 = 56.5%) came to our laboratory during a public event in the evening on a weekend between 6 p.m. and 1 a.m. where they were recruited and could immediately participate. The other sessions took part on weekdays between 9 a.m. and 6 p.m. For the morning sessions, the participants were instructed to have gotten-up at least two hours before the start of the experiment. Participants refrained from drinking (except water) and eating within at least one hour prior to the experiment.

**Fig 1:**
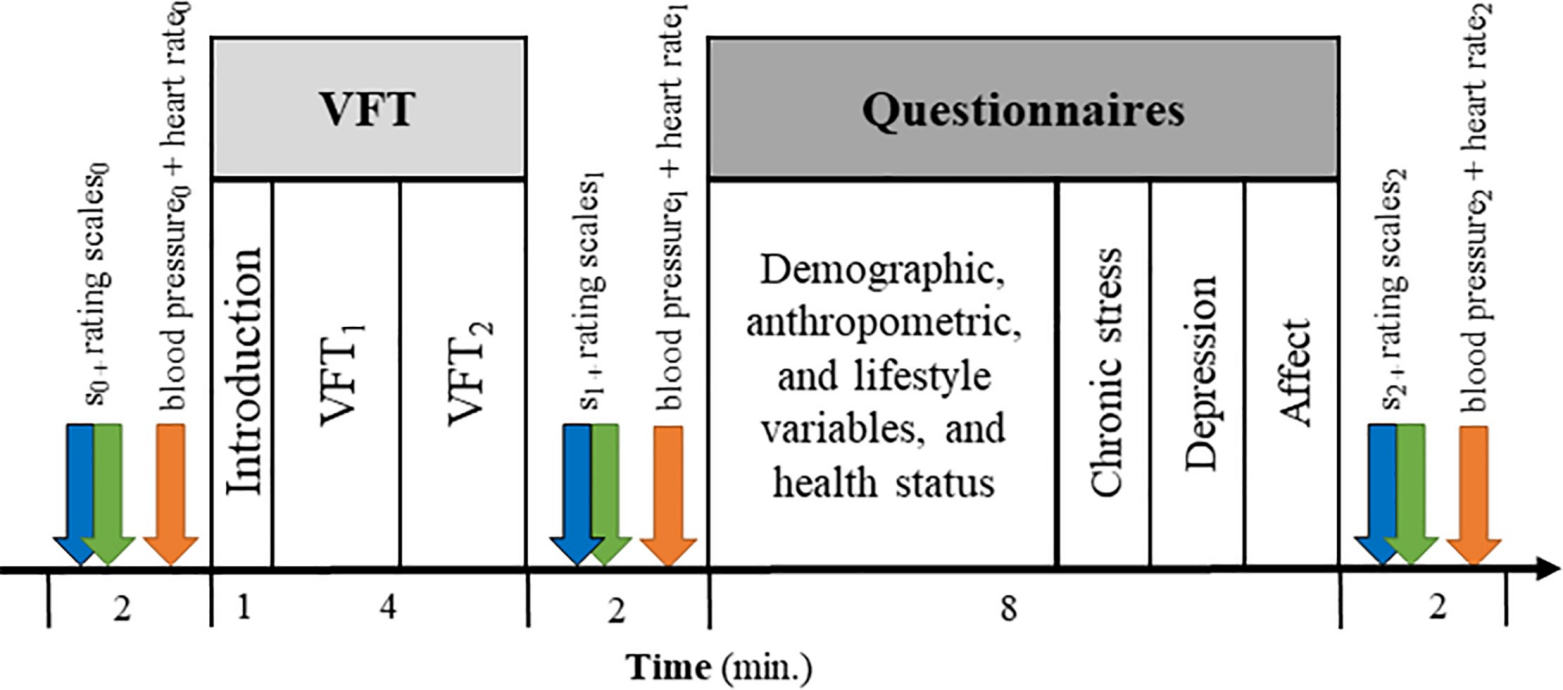
Time course of the experiment. Three saliva samples s_0_, s_1_, and s_2_ were collected at the time points t_0_, t_1_, and t_2_. The verbal fluency task (VFT) was performed between the first and the second saliva sample and consisted of two parts (VFT_1_ and VFT_2_). Furthermore, blood pressure was measured after each saliva sample.

Participants were seated in a comfortable chair in a quiet room. After they were informed about the experimental procedure, they gave their written consent for participation, and the first saliva sample (s_0_) was collected, and blood pressure and heart rate were measured for the first time. The VFT was then introduced to the participants and started immediately. The experimenters stayed in the room during the task. After this, the second saliva sample (s_1_) was collected and both blood pressure and heart rate were measured. To fill the gap between the second and third saliva collection, participants filled-out questionnaires, assessing their demographic variables, health status, depression, chronic life stress-perception, actual affect, and lifestyle factors. Ten minutes after s_1_, the last saliva sample (s_2_) was taken and blood pressure and heart rate were measured again. If necessary, participants were given more time to complete the questionnaires after s_2_. However, not everyone was willing to stay longer, so some questionnaire data is missing. Finally, weight and height were measured. During collection of the saliva samples, participants rated their actual perceived stress level, level of effort, and tiredness. The time course of the whole experiment is shown in Fig 1. The whole session lasted between 20 and 30 minutes, depending on the time needed for informing the participants and for filling-out the questionnaires.

### Statistical data analysis

For statistical analyses, IBM SPSS Statistics (version 26) was used. Normality of distribution was tested by means of the Kolmogorov-Smirnov test. Because of positive skewness and violation of normality, sAA levels were transformed by means of the square root transformation and cortisol levels by means of the natural logarithm prior to further statistical analysis. The data set that was used for statistical analysis and the corresponding codebook can be found in the S1 and S2 Files.

#### Main analyses

To test our main hypotheses (that the VFT leads to reactions of the SNS and the HPA axis as well as to changes in the stress, effort, and tiredness ratings), rmANOVAs with the within-subject factor ‘time’ (t_0_, t_1_, and t_2_) were calculated, separately for perceived stress ratings, sAA, and cortisol levels as well as for MAP and heart rate. For perceived stress ratings, the within-subject factor ‘state’ with the levels ‘stress’, ‘effort’, and ‘tiredness’ was included. To correct for multiple comparisons and because six separate rmANOVAs were calculated, an adjusted α-level of α_adjusted_ = α/6 = 0.05/6 = 0.008 was calculated according to the Bonferroni correction procedure [48]. Partial eta-squares (η_p_^2^) were considered as effect sizes. Sphericity was tested by means of the Mauchly test [49]. If necessary, degrees of freedom were corrected by means of the Greenhouse-Geisser procedure [50]. For post-hoc analysis, *t*-tests for dependent samples were calculated and Cohen’s *d* was considered as measure for effect sizes: *d* = (M_1_-M_2_)/σ with the means M_1_ and M_2_ and the standard deviation σ. For these dependent *t*-tests, Cohen’s *d* was corrected according to the method that was proposed by Morris (2008, [51]) after which the standard deviation σ is corrected as σ_corr_ = σ*[2*(1-r)]^1/2^, including the correlation *r* between variable 1 and 2.

#### Further and exploratory analyses

To investigate whether different orders in the VFT and the different categories that were used were associated with VF performance, a one-factorial ANOVA with the between-subjects factor ‘order’ (stress/animals, stress/foods, disease/animals, disease/foods, animals/stress, animals/disease, foods/stress, and foods/disease) was calculated. Furthermore, dependent *t*-tests were used to investigate whether performance differed between the neutral and emotional VFT as well as between the stress and disease and the animals and foods category.

For the exploratory analyses (i.e., to investigate whether the stress response or VFT performance, or the baseline levels were associated with age, sex, BMI, depression, chronic stress, or time of day), Pearson correlations were calculated. A Bonferroni-adjusted α-level of α_adjusted_ = 0.05/24 = 0.002 was used because 24 correlations were calculated (for sAA, cortisol, stress, effort, tiredness, MAP and heart rate at three time points, positive and negative affect, and VFT_perf_). Furthermore, it was examined whether differences in group means between depressed and non-depressed, high- and low-chronically stressed, and between hypertonic and non-hypertonic participants could be found. For this, independent *t*-tests with the same adjusted α-level of α_adjusted_ = 0.002 were calculated. Furthermore, it was investigated whether the physiological variables or VF performance differed between women that use oral contraceptives and women that do not by means of further independent *t*-tests. For these, an adjusted α-level of α_adjusted_ = 0.05/13 = 0.004 was used because 13 tests were performed (VFT performance and sAA, cortisol, MAP and heart rate at all three time points).

For further investigation of the associations between cortisol baseline levels and the cortisol time course, the variable ‘time of day’ was categorized into ‘early’ (before 12 p.m.), medium (12–18 p.m.), and late (after 18 p.m.). A further rmANOVA for cortisol was calculated with the within-subjects factor ‘time’ (t_0_, t_1_, and t_2_) and the between-subjects factor ‘time of day category’ (‘early’, ‘medium’, and ‘late’). Note: Unfortunately, it was not possible to investigate whether educational level was associated with VF performance because of too small group sizes.

## Results

### Verbal fluency performance

The mean VFT performance was VFT_perf_ = 26.9 ± 7.3 words (min.: 10.5, max: 48.5). Mean VFT performances for each category are summarized in Table 3. Mean VF performances did not differ between the different presentation orders and category combinations (stress/animals, stress/foods, disease/animals, disease/foods, animals/stress, animals/disease, foods/stress, and foods/disease; *F*_(7)_ = 0.36, *p* = .925). Verbal fluency performance did not differ between the stress and disease category (*t*_(83)_ = -1.88, *p* = .063, *d* = 0.38) as well as between the animals and foods category (*t*_(83)_ = -1.21, *p* = .230, *d* = 0.26). However, performance in the emotional VFT was significantly lower than in the neutral VFT (*t*_(84)_ = -12.1, *p* < .001, *d* = 1.51).

**Table 3:** Mean performances in the different verbal fluency tasks.

Category	Mean	Minimum	Maximum	Standard deviation
Stress	18.1	2	47	8.6
Disease	21.3	6	41	7.1
Animals	32.7	11	68	10.9
Foods	35.3	10	51	9.3

### Perceived stress ratings and affect

**Fig 2:**
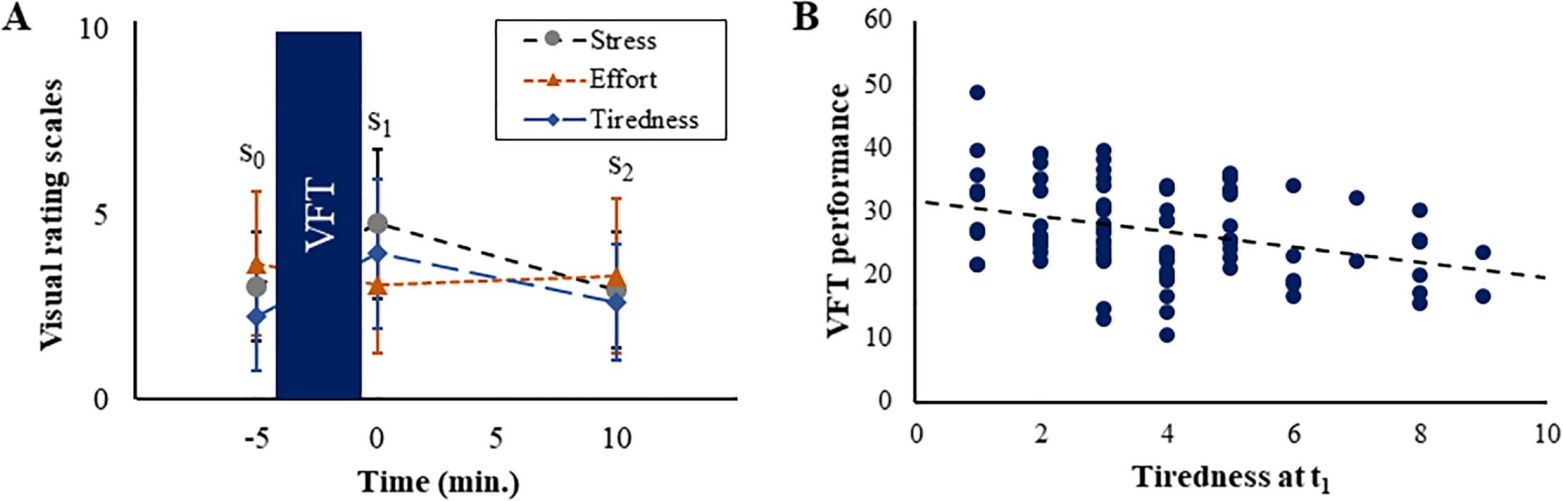
Ratings and significant association with task performance. (A) Time course of ratings for perceived stress, effort, and tiredness with respect to the verbal fluency task (VFT). Standard deviations are shown as error bars. (B) Association between VFT performance and tiredness at t_1_.

For the rating scales, a main effect of time (*F*_(2, 168)_ = 42.69, *p* < .001, η_p_^2^ = .34), a main effect of state (*F*_(1.45, 121.75)_ = 6.64, *p* = .005, η_p_^2^ = .07) and an interaction time x state (*F*_(3.0, 251.68)_ = 40.08, *p* < .001, η_p_^2^ = .32; Fig 2A) were found. Post-hoc rmANOVAs showed that all states significantly changed during the experiment, i.e. that a main effect of time was found (stress: *F*_(2, 168)_ = 56.00, *p* < .001, η_p_^2^ = .40, effort: *F*_(2, 168)_ = 7.17, *p* = .001, η_p_^2^ = .08, tiredness: *F*_(2, 168)_ = 48.50, *p* < .001, η_p_^2^ = .37). Post-hoc *t*-tests showed that perceived stress significantly increased between t_0_ and t_1_ (*t*_(84)_ = -8.61, *p* < .001, *d* = 1.00) and decreased between t_1_ and t_2_ (*t*_(84)_ = 9.80, *p* < .001, *d* = 1.01). Effort ratings significantly increased between t_0_ and t_1_ (*t*_(84)_ = 4.30, *p* < .001, *d* = 0.46). Tiredness significantly increased between t_0_ and t_1_ (*t*_(84)_ = -9.60, *p* < .001, *d* = 1.32) and decreased between t_1_ and t_2_ (*t*_(84_) = 6.40, *p* < .001, *d* = 0.82). Only tiredness at t_1_ was correlated with task performance (*r*_(84)_ = -.33, *p* = .002; Fig 2B). None of the other ratings was associated with VFT performance after correction for multiple comparisons (all *p* ≥ .044). The mean values of the ratings are provided in Table 4.

**Table 4:** Mean values and standard deviations of the perceived ratings and the scales of the Positive and Negative Affect Schedule (PANAS).

	N	Mean	Standard deviation	Minimum	Maximum
Stress t_0_	85	3.1	1.8	1	8
Stress t_1_	85	4.7	2.0	1	9
Stress t_2_	85	3.0	1.7	1	8
Effort t_0_	85	3.7	2.0	1	8
Effort t_1_	85	3.1	1.8	1	8
Effort t_2_	85	3.3	2.1	1	10
Tiredness t_0_	85	2.2	1.5	1	7
Tiredness t_1_	85	3.9	2.0	1	9
Tiredness t_2_	85	2.6	1.6	1	9
PANAS positive	78	3.0	0.7	1.30	4.30
PANAS negative	78	1.4	0.4	0.90	3.30

Immediately before t_2_, the PANAS was filled-out by the participants. At this time point, positive affect was significantly higher than negative affect (*t*_(77)_ = 16.35, *p* < .001, *d* = 1.5, M_positive_ = 3.03 ± 0.71, M_negative_ = 1.38 ± 0.41). After correction for multiple comparisons, a marginally significant negative correlation between negative affect and VFT performance was found (*r*_(76)_ = -.27, *p* = .017). Neither positive nor negative affect was related with any of the other physiological or psychological variables at t_2_ (all *p* ≥ .053).

### Alpha-amylase

**Fig 3:**
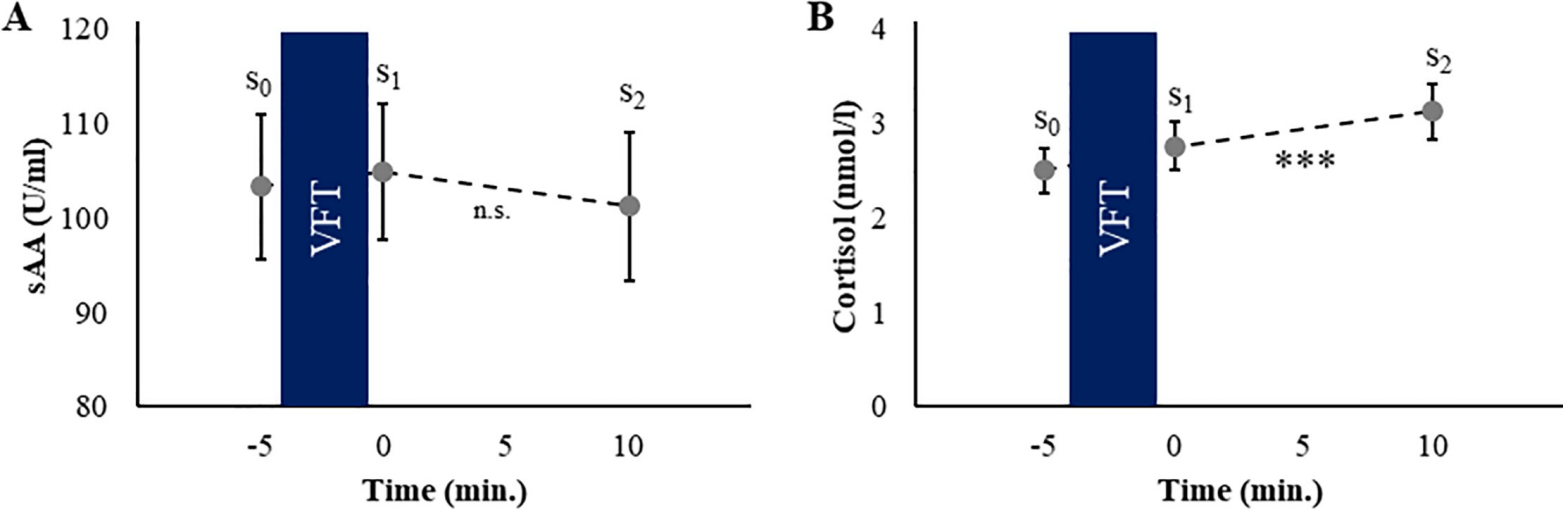
Alpha-amylase and cortisol responses. (A) Time course of salivary α-amylase (sAA) with respect to the verbal fluency task (VFT). (B) Cortisol time course. Standard errors are shown as error bars.

Salivary α-amylase levels did not significantly differ between the three measurement-time points (*F*_(1.8, 150.0)_ = 0.69, *p* = .487, η_p_^2^ = .008; Fig 3A). Salivary α-amylase levels were not associated with VFT performance (all *p* ≥ .293). When only including the non-hypertonic participants into the analysis, no main effect of time was found (*F*_(1.8, 101.9)_ = 3.07, *p* = .057, η_p_^2^ = .05). No associations with VF performance were found as well (all *p* ≥ .278). An overview of the mean sAA levels and of all other physiological variables at all three time points is provided in Table 5.

**Table 5:** Mean values and standard deviations of the physiological variables.

	N	Mean	Standard deviation	Minimum	Maximum
Cortisol t_0_ (nmol/l)	85	2.5	2.2	0.3	10.7
Cortisol t_1_ (nmol/l)	85	2.8	2.4	0.5	12.8
Cortisol t_2_ (nmol/l)	85	3.1	2.7	0.5	13.8
sAA t_0_ (U/ml)	85	103.3	71.0	3.9	331.2
sAA t_1_ (U/ml)	85	104.9	66.3	5.3	302.8
sAA t_2_ (U/ml)	85	101.2	72.5	1.9	306.3
Systole t_0_ (mmHg)	84	132.1	18.1	82	192
Systole t_1_ (mmHg)	83	130.1	16.6	102	178
Systole t_2_ (mmHg)	83	127.6	15.5	104	179
Diastole t_0_ (mmHg)	83	83.6	9.2	63	118
Diastole t_1_ (mmHg)	83	82.7	9.7	61	108
Diastole t_2_ (mmHg)	83	82.0	10.4	50	104
MAP t_0_ (mmHg)	83	103.4	11.7	70.8	136.7
MAP t_1_ (mmHg)	83	102.2	11.2	80.2	129.4
MAP t_2_ (mmHg)	83	100.8	11.6	73.9	128.4
Heart rate t_0_ (bpm)	83	75.1	12.3	51	111
Heart rate t_1_ (bpm)	83	73.4	11.7	47	105
Heart rate t_2_ (bpm)	83	72.5	10.6	46	99

sAA: salivary α-amylase; MAP: mean arterial pressure; bpm: beats per minute.

### Cortisol

For cortisol, a main effect of the factor time was found (*F*_(2, 168)_ = 19.29, *p* < .001, η_p_^2^ = .19, Fig 3B). Post-hoc analyses showed that cortisol levels significantly increased between t_0_ and t_1_ (*t*_(84)_ = -3.58, *p* = .001, *d* = 1.48) and increased further between t_1_ and t_2_ (*t*_(84)_ = -4.0, *p* < .001, *d* = 0.44). However, cortisol levels were not associated with VFT performance (all *p* ≥ .370).

### Blood pressure and heart rate

**Fig 4:**
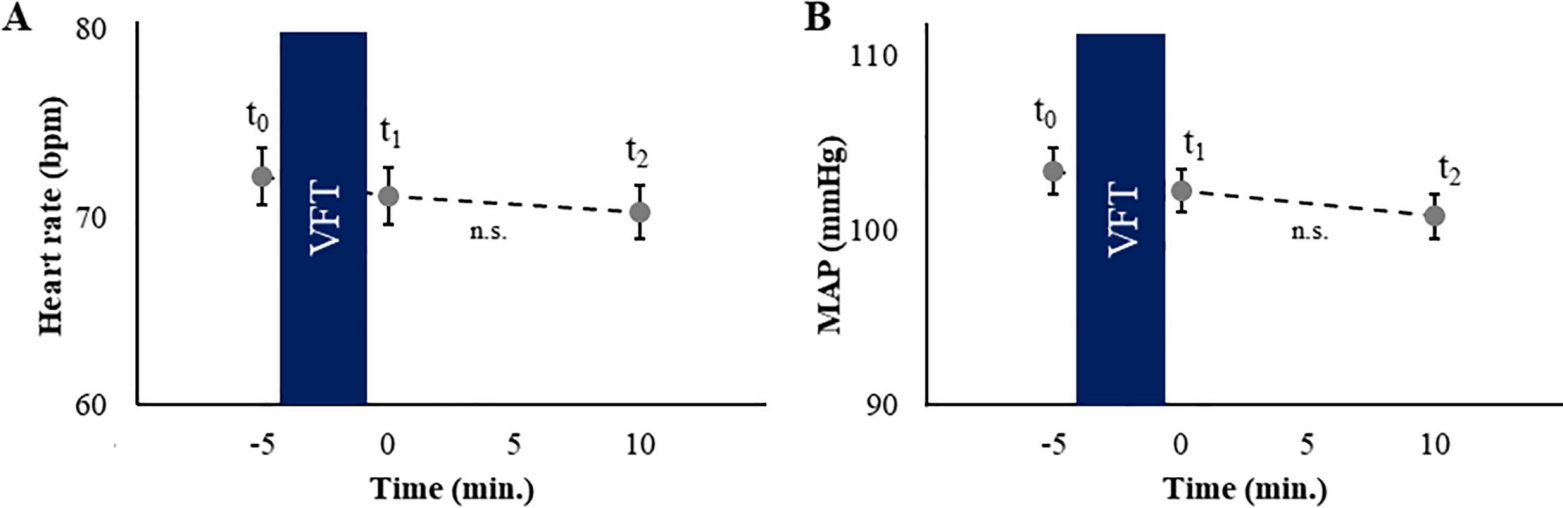
Heart rate and mean arterial pressure response. (A) Time course of the heart rate in beats per minute (bpm) with respect to the verbal fluency task (VFT). (B) Time course of the mean arterial pressure (MAP). Standard errors are shown as error bars.

When all participants were included into the analysis, a main effect of the factor time was found (*F*_(2, 159.8)_ = 6.36, *p* = .002, η_p_^2^ = .06, Fig 4A). Post-hoc *t*-tests showed an marginal significant decrease in heart rate between t_0_ and t_1_ (*t*_(82)_ = 2.17, *p* = .033, *d* = 0.23). Heart rate was not associated with VFT performance (all *p* > .288). When only the non-hypertonic participants were included into the analyses, no effects were found (all *p* ≥ .069).

When all participants were included into the analysis, no main effect of the factor time was found for MAP (*p* = .04). No associations between MAP and VFT performance were found, neither for the whole sample nor for the non-hypertonic participants (all *p* ≥ .103).

### Exploratory analysis

To investigate whether anthropometric and health factors (e.g., age, sex, BMI, chronic stress, depression, and use of oral contraceptives) which are typically related with HPA axis and SNS activity and responses to acute stressors when other stress tasks are used (e.g., [29–33,35]) are related with the stress response or with VF performance in our study, exploratory analyses were conducted. Furthermore, it was investigated whether the time of day was related with the stress response or with the VF performance, because a part of the experiment was conducted during a public event in the evening, and the rest was carried out in the morning or the afternoon.

#### Anthropometric variables

First, it was investigated whether the stress response in our experiment was associated with the factors age, sex, and BMI that are known to be typically related with the stress response [30–32]. However, no associations were found when using the adjusted α-level of α_adj_ = .002 (all *p* ≥ .003).

#### Use of oral contraceptives

Next, it was investigated whether VF performance or the physiological variables differed between women who use oral contraceptives and women who do not because it has been shown previously that oral contraceptives might affect stress axes activity [35]. However, no differences were found (all *p* ≥ .208).

#### Depression

Since it has been shown previously that the stress response and cognitive performance can be associated with depression (e.g., [33,52]), it was investigated whether this can be found in our study as well. Correlation analyses indicated that neither the physiological variables nor the cognitive performance were associated with depression (all *p* ≥ .113). Only negative affect and level of effort at t_0_ were related with depression (negative affect: *r*_(78)_ = .45, *p* < .001, effort at t_0_: *r*_(78)_ = .38, *p* = .001). After classifying the participants into depressed and non-depressed according to the cut-off value by Stein and Luppa (2012; [36]), only a difference in negative affect was found between the groups (*t*_(75_) = -4.25, *p* < .001, *d* = 1.4, M_non-depressed_ = 1.3 ± 0.32, M_depressed_ = 1.9 ± 0.63). Neither the physiological variables nor the other measures differed between depressed and non-depressed participants (all *p* > .014).

#### Chronic stress

Next, it was investigated whether the stress response was associated with the level of perceived life stress during the last month because it is well known that chronic stress can alter stress axis activity (e.g., [53,54]). Perceived stress at t_0_ and t_1_ as well as positive and negative affect were related with the PSS score (perceived stress at t_0_: *r*_(83)_ = .33, *p* = .002, perceived stress at t_1_: *r*_(83)_ = .34, *p* = .002, positive affect: *r*_(76)_ = -.31, *p* = .001; negative affect: *r*_(76)_ = .51, *p* < .001). Furthermore, a marginally significant association between VFT performance and PSS scores was found (*r*_(83)_ = .28, *p* = .008). None of the physiological variables were related to PSS levels (all *p* ≥ .105). After classifying the participants into low- and high-chronically stressed according to the cut-off values by Klein et al. (2016; [44]), a significant difference for positive affect (*t*_(76_) = 3.53, *p* < .001, *d* = 0.77; M_low_ = 3.3 ± 0.62, M_high_ = 2.8 ± 0.69) and a marginal difference in perceived stress at t_1_ (*t*_(83_) = -2.73, *p* = .008, *d* = 0.6; M_low_ = 4.1 ± 1.86, M_high_ = 5.2 ± 1.86) was found between the low- and the high-chronically stressed participants.

#### Time of day

At last, it was analyzed whether time of day was associated with task performance, baseline levels, or with the stress response. All cortisol levels were associated with time of day (t_0_: *r*_(83)_ = -.54, *p* < .001, t_1_: *r*_(83)_ = -.50, *p* < .001, t_2_: *r*_(83)_ = -.42, *p* < .001, However, the percentage of the cortisol increase (between t_0_ and t_2_ (i.e., the HPA axis response) which was calculated as cort_increase_ = [cort_t2_ –cort_t0_]*100/cort_t0_, and therefore the stress response, was not related with time of day (*p* = .052). With a further rmANOVA, only main effects of the within-subject factor time (*F*_(1.38, 113.23)_ = 9.88, *p* = .001, η_p_^2^ = .11) and the between-subjects factor ‘time of day-category’ (*F*_(2, 82)_ = 13.74, *p* < .001, η_p_^2^ = .25), but no interaction time x time of day-category was found (*p* = .169). No associations were found for the other variables (neither for the physiological nor the perceived ratings nor for VF performance; all *p* > .003).

## Discussion

The aim of our study was to investigate whether a VFT induces an acute stress response, i.e. if it leads to an increase in the activity of the SNS and the HPA axis and to an increase in perceived stress. Furthermore, we aimed to investigate whether–if an acute stress response was found–this was associated with VF performance. We hypothesized that higher stress responses would be related with poorer performance.

As expected, an increase of cortisol levels and, therefore, an increase in HPA axis activity was found. This was not related with VF performance. Furthermore, levels of perceived stress and effort as well as tiredness were higher immediately after the VFT than before. However, changes in HPA axis activity and ratings of perceived stress and effort were not associated with cognitive performance during the task. Only tiredness immediately after the task was negatively associated with task performance. No changes in SNS activity were found at all, neither in sAA levels, nor in blood pressure, nor in heart rate.

Associations between HPA axis activity and VF performance have been reported previously. Greendale, Kritz-Silverstein, Seeman, and Barrett-Connor (2000; [55]) found that basal cortisol levels predicted VF performance four years later, i.e. higher cortisol levels were associated with worse verbal fluency performance. Fiocco, Joober, and Lupien (2007; [56]) reported that participants with lower educational levels (which might be associated with poorer VF performance) showed higher cortisol responses to a TSST and performed worse in a VFT than participants with higher educational levels. Unfortunately, we were not able to investigate whether educational level was associated with the VF performance or with the HPA axis response in our study because of too small group sizes.

Numerous stress induction tasks have been developed so far such as the TSST [24,57] or the Montreal Imaging Stress Task (MIST; [58]) which include combinations of social-evaluative and cognitive stressors, or the socially evaluated cold-pressor test (SECPT; [59–61]) which involves a physiological and a social-evaluative stress component. The speciality in our study was that we used a purely cognitive stressor without inducing a social-evaluative component. Other cognitive stressors (e.g., mathematical [62,63] or inhibition tasks such as the Stroop task; [64]) have been proposed previously and have been shown to induce subjective and physiological stress responses as well. However, to the best of our knowledge we were the first who have shown that a VFT can be a suitable alternative, especially when HPA axis and perceived stress responses are intended.

At first glance, it is astonishing that no response of the SNS could be found in our study, because usually this is a reliable, quickly activated stress indicator. Therefore, it might have been that our SNS markers were not sensitive enough to detect changes in response to the VFT and that we should have used alternatives such as blood samples of epinephrine or norepinephrine, electrodermal activity, or heart rate variability. However, if our finding could be replicated in future research, this would have interesting theoretical implications that fit well into modern stress theories. In contrast to the widespread view that there is a general stress response that is independent of the type of the stressor (so-called generality model; [2]), the integrated specificity model [25] is becoming increasingly popular. The main assumption of this model is that the stress response is specific and that it depends on the stressor characteristics, e.g. whether the stressor is threatening or challenging, emotional, social-evaluative, or cognitive. According to the integrated specificity model, different physiological reactions occur, depending on the stressor‘s properties. Social-evaluative stressors, for example, which elicit the emotion shame, lead to strong HPA axis responses (so-called Social-Self Preservation Theory; [65]). Cognitive stressors have been shown to elicit HPA axis responses as well [66] which can be amplified when combined with social-evaluative stressors. In contrast, threatening and emotional stressors have been shown to elicit strong SNS responses [2]. Therefore, our results fit well into this modern approach of stress specificity.

Overall, we suggest that a VFT can be an easy to implement alternative to other laboratory stress tasks that use pure cognitive stressors. This has the further advantage that cognitive performance can be measured at the same time as the stress induction. However, we cannot completely rule out that we created a social-evaluative component accidentally. This could have happened through the dictaphone that was placed in front of the participants or through the presence of our assistants. In future research, it should therefore be investigated whether a VFT without the presence of an experimenter and without a (visible) dictaphone leads to the same effects.

Our results have some practical implications because VFT are a standard procedure in neuropsychological assessments. An HPA axis activation after this test could affect subsequent physiological measurements as well as performance in subsequent cognitive assessments. However, this is a problem that cannot be ruled out and that is likely to occur in all cognitive assessments.

A number of additional exploratory analyses were performed to investigate whether associations can be found with anthropometric and health factors (e.g., age, sex, BMI, chronic stress, depression, and use of oral contraceptives) which are typically related with HPA axis and SNS activity or cognitive performance [29–33,35]. However, no associations between the stress response or VF performance and these variables were found. Therefore, we conclude that it is not necessary to include them as control variables in future studies when the same task is used. However, future research is needed to replicate this lack of findings.

The main limitation of our study might be that a part of it was conducted in the late evening until midnight which is not typical for laboratory settings. Due to the circadian rhythm of the HPA axis, cortisol levels are lower in the evening than at earlier times of the day [53,67]. This was also found in our study. However, no association between the HPA axis response (i.e., the percentual cortisol increase) and time of day was found. A significant cortisol increase was found independently of the baseline cortisol level and independently of the time of day. Furthermore, the large range in assessment times could also be seen as an advantage because it enabled us to recruit a variety of participants. Furthermore, clinical assessments in which the VFT is usually used also take place at different times of the day. Our study is subject to some further limitations that should be addressed in future research. Most importantly, other physiological variables (e.g., heart rate variability, blood samples of epinephrine and norepinephrine, and electrodermal activity) should be measured to verify that indeed no SNS activation has been induced. Furthermore, brain activity (e.g., assessed by means of electroencephalography recordings) and its association with the stress response should be investigated as an additional measure to VF performance to evaluate whether different cognitive demands (and, therefore, different neural processes) are associated with the stress response. Furthermore, longer time intervals should be used in future research to assess the time point and peak amplitude of the maximal cortisol increase as well as HPA axis recovery. Besides, other control variables which have been shown to be related to HPA axis responses or to VF performance (e.g., education, childhood traumata, general cognitive performance, physical health, or neurological diseases in which cognitive performance is impaired; e.g. [56,68,69]) should be included in future studies. Additionally, sex hormones should be assessed in future studies because the HPA axis and the hypothalamic-pituitary-gonadal axis could interact which typically leads to stronger HPA axis responses in men than in women [29,70,71]. This might have influenced our results as well, although no sex differences were found. The assessment of the participants’ sex by means of questionnaires only might have not been enough. Moreover, we did not control for animal anxiety or phobia or for mental or physical problems related to food which might have affected VF performance in the respective categories. Therefore, either this should be assessed in future studies or other neutral categories should be used. Last, further variations of the VFT should be investigated in future research such as using a phonemic fluency task, using only emotional or neutral categories, or using more categories.

## Conclusions

We conclude that a VFT is an acute stressor that induces a cortisol response without the need of further (e.g., social-evaluative) stress components. Therefore, it is a less time-consuming alternative to other stress tasks that can be used in field studies without much effort. Furthermore, our results fit well into the integrated specificity model and do not support the view of a general, unspecific stress response. However, the lack of an SNS response must be replicated in future studies.

## Supporting information

S1 File (XLSX)

S2 File (XLSX)
